# Abstinence Rate, Adverse Events and Withdrawal Symptoms after Varenicline Use and Predicting Factors of Smoking Abstinence: A Multicentre Single-State Study in Malaysia

**DOI:** 10.3390/ijerph19137757

**Published:** 2022-06-24

**Authors:** Shea Jiun Choo, Chee Tao Chang, Balamurugan Tangiisuran, Mohd Faiz Abdul Latif, Nor Aida Sanusi, Sabariah Noor Harun

**Affiliations:** 1 School of Pharmaceutical Sciences, Universiti Sains Malaysia, Gelugor 11800, Malaysia; bala@usm.my (B.T.); sabariahnoor@usm.my (S.N.H.); 2 Pharmacy Department, Hospital Taiping, Ministry of Health Malaysia, Taiping 34000, Malaysia; 3 Clinical Research Centre, Hospital Raja Permaisuri Bainun, Ministry of Health Malaysia, Ipoh 30450, Malaysia; 4 Taiping Health Clinic, Ministry of Health Malaysia, Taiping 34000, Malaysia; thefaizlatif17@gmail.com; 5 Perak Pharmaceutical Services Division, Ministry of Health Malaysia, Ipoh 31250, Malaysia; nstkc92@yahoo.com

**Keywords:** varenicline, smoking cessation, treatment outcome, Malaysia

## Abstract

(1) Background: Varenicline is a widely prescribed agent in smoking cessation. However, the abstinence rate, the incidence of adverse events and withdrawal symptoms, have not been widely studied locally. This study aimed to determine the prevalence of smoking abstinence, adverse events and withdrawal symptoms associated with varenicline use, as well as possible factors contributing to successful smoking abstinence. (2) Methods: This was a retrospective, cohort study conducted in twenty-two government-operated smoking cessation clinics across the state of Perak, Malaysia. The medical records of adult smokers (age ≥ 18 years old) who were prescribed with varenicline between January 2017 and June 2018 were traced. The medical records of smokers who used pharmacotherapy other than varenicline, those who received less than four weeks of varenicline treatment, and with missing data were excluded. (3) Results: Sixty-eight out of 114 subjects (59.6%) successfully achieved smoking abstinence. Probable varenicline-induced chest pain was documented in three subjects. Altered behaviour (*n* = 2) and auditory hallucinations (*n* = 1) were also reported. Varenicline treatment duration is a significant predictive factor for successful smoking abstinence (odds ratio (OR) = 2.45; 95% confidence interval (CI) 1.74–3.45; *p* < 0.001), followed by age (OR = 1.25; 95% CI 1.005–1.564; *p* = 0.045), the presence of adverse events (OR = 0.096; 95% CI 0.014–0.644; *p* = 0.016) and withdrawal symptoms (OR = 0.032; 95% CI 0.016–0.835; *p* = 0.032). (4) Conclusion: Almost two-thirds of the subjects achieved smoking abstinence with varenicline. The duration of the treatment, as well as the patients’ ages had a significant influence on successful smoking abstinence. Rare cases of cardiovascular and neuropsychiatric-related adverse events were reported, warranting continuous surveillance and adverse drug reaction reporting.

## 1. Introduction

Smoking remains a major public health problem in Malaysia. The prevalence of smokers in Malaysia was 22.8 percent in 2015 [1], and it was estimated that nearly 5.3 million Malaysians ages 15 years and older smoked [2]. Malaysia needs to reduce the prevalence of smoking to 15 percent by the year 2025 in order to achieve the World Health Organisation’s (WHO) non-communicable diseases global target [3]. However, Malaysia is projected to fail to reach the global target for reducing tobacco use [2]. Based on the Malaysian Global Adult Tobacco Survey (GATS) report in 2011, only nine percent of the smokers used pharmacotherapy in their attempts to quit. The majority attempted to quit without assistance and proper guidance, which might contribute to the low smoking cessation rate [4]. Hence, expanding the coverage of services that help people quit smoking is one of the approaches taken by the health authority to promote successful smoking cessation [4].

Varenicline is a partial agonist and blocks the nicotinic acetylcholine receptor, inhibits nicotine stimulation of the dopaminergic system [5]. In 2011, varenicline was introduced and approved for smoking cessation by the Malaysian Ministry of Health. As an alternative to nicotine replacement therapy (NRT), varenicline is prescribed to smokers who are treated under the government-operated smoking cessation clinics. Previous studies showed that varenicline has more favourable odds in achieving abstinence of six months or longer when compared to NRT monotherapy and bupropion [6,7,8].

Despite varenicline’s superiority, the U.S. Food and Drug Administration (FDA) issued a public health advisory to alert smokers, caregivers and healthcare professionals about the risks of varenicline causing neuropsychiatric and cardiovascular adverse events, as demonstrated in 2008 and 2011, respectively [9,10]. The FDA warning was subsequently removed based on the findings from later studies in 2012 and 2016 [11,12]. Nevertheless, healthcare professionals are constantly reminded to monitor patients for neuropsychiatric and cardiovascular adverse events and to weigh the risks against the benefits when prescribing varenicline.

Varenicline is a widely prescribed agent in the government-run smoking cessation clinic, also known as the Quit Smoking Clinic in Malaysia. Within the local context, the choice of nicotine replacement therapies or varenicline was primarily guided by the National Clinical Practice Guidelines for Treatment of Tobacco Use Disorder [13], followed by its availability, efficacy, safety, suitability and cost, and the final decision was made based on consensus between the clinician and patient. However, the abstinence rate, and the incidence of adverse events and withdrawal symptoms, as well as smoker characteristics related to successful smoking cessation with varenicline use, have not been widely studied locally. Moreover, no study has been done to determine the association between the occurrence of adverse events and withdrawal symptoms and smoking cessation outcomes. This study aimed to determine the prevalence of smoking abstinence, adverse events and withdrawal symptoms associated with varenicline use, as well as possible factors contributing to successful smoking abstinence. 

## 2. Materials and Methods

### 2.1. Study Design and Population

This was an observational, retrospective cohort study, conducted in 22 government-operated smoking cessation clinics covering seven out of 12 districts in the Perak state of Malaysia. The study population comprised smokers who registered and followed up at the selected government-operated quit smoking clinics, which included those who were referred for smoking cessation and walk-in smokers who wished to quit voluntarily. 

We used The National Clinical Practice Guidelines for Treatment of Tobacco Use Disorder as a guide for patient recruitment into the varenicline treatment [13]. Briefly, all patients who first visited the quit smoking clinics would be asked about their smoking status, including the number of cigarettes smoked per day and previous attempts to quit. Subsequently, advice to quit and assessment on the willingness to make a quit attempt was performed by the clinician. For patients who were willing to quit, counselling would be provided on the available pharmacotherapy agents, and follow-up was scheduled within the first week after the quit date. For patients who were not ready to quit, a motivational interview was conducted to explore reasons for unwillingness to quit, and explaining risks and rewards of quitting smoking.

All medical records of adult smokers (age ≥ 18 years old) who were prescribed varenicline as pharmacotherapy for smoking cessation between January 2017 and June 2018 were traced. The medical records (including abstinence and adverse events) were traced according to the quit smoking clinic visits schedule, at a bi-weekly time-point during the first 12 weeks (Week-2, 4, 6, 8, 10, 12) and four-weekly thereafter (Week-16, 20, 24). Medical records of smokers who used pharmacotherapy other than varenicline for smoking cessation, of those who received less than four weeks of varenicline treatment, and of those who had missing data were excluded. 

The sample size was calculated using a Raosoft sample size calculator. Based on an abstinence rate of 47 percent [14], with a five percent margin of error and a 95 percent confidence level, the calculated sample size needed was 150 subjects. 

### 2.2. Investigated Variables

Demographic characteristics of the subjects, along with their smoking history and behaviour, were extracted from the medical records into a standardised data collection form derived from variables in the smoking cessation clinic record booklet. Data on cardiovascular, neuropsychiatric-related and other adverse events, as well as withdrawal symptoms, were collected based on the doctor’s documentation in the follow-up booklet at the smoking cessation clinic. 

The prevalence of smoking abstinence was derived by dividing the number of smokers who received varenicline and achieved smoking abstinence (self-reported, at least 2 weeks) by the total number of smokers receiving varenicline treatment [15]. Continuous abstinence was defined as the length of abstinence between quit time and a follow-up date [16]. 

The number of cigarettes smoked per day, the age the patients started smoking and their attempts to quit smoking were based on the smokers’ self-reporting during their visits at the clinic. The Fagerstrom score was used to assess the intensity of their physical addiction to nicotine, with 0–3 being low dependence, 4–5 being moderate dependence and 6–10 being high dependence [17].

Readiness to quit comprised four stages: (i) precontemplation: not planning to quit smoking within the next six months; (ii) contemplation: considering quitting within the next six months; (iii) preparation: planning to quit within the next 30 days; and (iv) action: having successfully quit for less than six months. Those at the precontemplation and contemplation stages were classified as not ready to quit, while those at the preparation and action stages were categorised as ready to quit.

### 2.3. Statistical Analysis

All the data collected were analysed using SPSS version 24 (Armonyx, NY, USA). Demographic characteristics, smoking behaviour and history, and abstinence rate, as well as incidence of adverse events and withdrawal symptoms, were analysed using descriptive analysis, presented in frequencies and percentages for categorical variables. Continuous data were described as mean and standard deviation. The chi-squared test or Fisher’s exact test was used to explore associations between varenicline treatment outcomes and smokers’ characteristics. Predictive effects of demographic characteristics and smokers’ behaviour and history, which may contribute to smoking abstinence, were analysed using univariate and multiple logistic regression. Variables with *p* < 0.25 in univariate analysis were entered into the multivariate regression model. The level of statistical significance for all the data presented was set at *p* < 0.05. 

## 3. Results

### 3.1. Smoking Abstinence

A total of 245 medical records of smokers prescribed with varenicline for smoking cessation were screened. The 103 smokers who received varenicline for less than four weeks and 28 smokers with missing data were excluded. Eventually, a total of 114 smokers were included in the final analysis. Most of the smokers were male (*n* = 104, 91.2%), and more than half were Malays (*n* = 70, 61.4%). The mean age of the smokers was 46 ± 14.4 years, ranging from 21 to 78 years old. Most of the smokers had at least one comorbid condition (*n* = 69, 60.5%) and 39.5% (*n* = 45) reported no known medical illness. The most prevalent comorbidities were diabetes mellitus, followed by hypertension and dyslipidaemia (Table 1).

The mean duration of smoking was 26.6 ± 14.9 years. The number of smokers with high nicotine dependence (*n* = 44, 38.6%) was comparable to that of those with low nicotine dependence (*n* = 42, 36.8%). More than half of the smokers were prepared and took action to quit (*n* = 81, 71%), whereby most had attempted smoking cessation (*n* = 79, 69.3%). Situational factor (after meal; *n* = 89, 78.1%) was the most common trigger that stimulated smoking behaviour, followed by mood effect (feeling bored/drowsy; *n* = 84, 73.7%), peer influence (when colleagues/friends are smoking; *n* = 82, 71.9%), anxiety/stress (*n* = 63, 55.3%) and a social event or a festive season (*n* = 57, 50%) (Table 2). 

The mean treatment duration among the varenicline users was 8.8 ± 3.3 weeks. The prevalence of smoking abstinence with varenicline therapy was 59.6% (*n* = 68). Amongst smokers who successfully quit, only 18.4% (*n* = 13) achieved continuous abstinence for 24 weeks (Table 3).

### 3.2. Adverse Events and Withdrawal Symptoms

Generally, varenicline was well tolerated among the users. Out of 114 smokers taking varenicline, 65 (57.0%) did not experience any adverse effects. The most common adverse events reported were increased appetite (*n* = 19, 16.7%), dizziness (*n* = 18, 15.8%) and dry mouth (*n* = 12, 10.5%). Notably, two smokers refused to continue varenicline therapy due to nausea and insomnia intolerance, respectively (Table 4). Three smokers developed chest pain after taking varenicline, and two of them required hospitalization. Regarding neuropsychiatric-related adverse events, two smokers were documented to have altered behaviour, and one had auditory hallucinations. Otherwise, there was no incidence of depression, seizure, suicidal attempt/ideation, or completed suicide. Varenicline therapy was discontinued for all smokers with cardiovascular and neuropsychiatric-related adverse events. Forty-five smokers experienced withdrawal symptoms. The top five frequently reported withdrawal symptoms among the smokers were fatigue (*n* = 21, 18.4%), headache (*n* = 18, 15.8%), irritability/restlessness (*n* = 15, 13.2%), anger/frustration (*n* = 14, 12.3%) and change in sleep pattern (*n* = 12, 10.5%) (Table 4).

### 3.3. Factors Associated with Successful Abstinence

Overall, 4 variables obtained a *p*-value of less than 0.25, i.e., race, comorbidity status, presence of adverse events and varenicline treatment duration. These variables were entered into the multivariate regression model. An additional 1 week of varenicline treatment duration is associated with nearly 1.8 times the odds of success in smoking cessation (OR 1.83; 95% CI 1.50–2.23; *p* < 0.001). No other significant predictors were found (Table 5). Interactions, multicollinearity and suppressor effects were checked and not found [18]. 

## 4. Discussion

To the best of our knowledge, this is amongst the first multicentre studies to study the prevalence of smoking abstinence among varenicline users in Malaysia. Most of the subjects successfully achieved smoking abstinence with varenicline use, while other local studies that used both NRT and varenicline demonstrated a relatively lower abstinence rate [19,20]. Compared to other regions of government-operated quit smoking clinics, the current study reported lower rates of smoking abstinence in the third month in Perak (19.3%) versus in Putrajaya and Selangor (35.4%) [21] and lower rates of smoking abstinence in the sixth month in Perak compared to a health clinic in Kuala Lumpur (18.4% vs. 50.5%) [22]. This might be attributed to the difference in income and educational status between the study population, where smokers with higher education and income reported higher odds of abstinence [23].

The current study shows that older age and the absence of underlying diseases are not significant factors associated with successfully quitting smoking. In contrast, a large-scale study in China found that older age was associated with higher odds of smoking abstinence [24]. Meanwhile, Joly et al. found that smoking-related diseases do not facilitate smoking cessation or the maintenance of abstinence, as smokers with comorbidities may be discouraged from stopping since the diseases have already occurred [25]. 

Varenicline treatment duration was notably a significant determinant of successful quit attempts in this study. The recommended duration of varenicline therapy as per prescribing information is 12 weeks. However, the mean duration of treatment reported in this study was approximately one month shorter. This could be due to the smokers’ early and high dropout rate from the smoking cessation programme [20]. Therefore, a targeted smoking cessation approach could be implemented, where the counselling should emphasize adherence to completion of the treatment regimen. 

Smokers’ experiences of adverse events and withdrawal symptoms were reported to be associated with their maintenance of the smoking cessation pharmacotherapy, causing a failure to quit smoking [26,27]. Nevertheless, the presence of adverse events or withdrawal symptoms did not influence the odds of quitting smoking in this study. Two subjects opted for discontinuation of varenicline therapy despite minor and self-limiting adverse events. In a local qualitative study conducted by Chean et al., smokers highlighted withdrawal symptoms such as lethargy and constipation as barriers to quitting [28].

Psychosocial characteristics, such as motivation [25,29,30] and a high level of confidence [31,32], may interfere with the result of smoking cessation attempts. The frequency of success was four times higher when the smokers’ readiness to quit escalated from the “precontemplation” stage to the “contemplation” stage and two times higher from the “preparation” stage to the “action” stage [25]. Nevertheless, our study did not discover this relationship, most likely due to its small sample size. Since smokers at the precontemplation stage are unlikely to quit [29], approaches such as education about the positive outcomes of smoking cessation and the identification of misconceptions and barriers to quitting may encourage change in readiness to quit. On the contrary, Ussher et al. argued that the motivation to stop smoking only predicts the incidence of quit attempts, not the success of those attempts [33]. Future interventions may focus on facilitating quit attempts by employing trained clinical pharmacists to address the smokers’ urge to smoke, rather than solely assessing and boosting motivation towards quitting [34,35].

In the United States, the use of varenicline for 24 weeks elicited a continuous abstinence rate of 37.8%, while serious adverse events occurred in 3.7% of the varenicline users and 0.8% presented with suicidal ideation [36]. Meanwhile, a global study across Latin America, Africa and the Middle East reported a continuous abstinence rate of 39.7% at 24 weeks, and the severe adverse events were reported in 2.8% of varenicline recipients, including suicidal ideation and abnormal heart rate [37]. Likewise, Rennard and colleagues reported a continuous abstinence rate of 34.7% at week-24, with a serious adverse events rate of 1.2%, including carotid artery stenosis and peripheral arterial occlusive disease [38]. An Asian trial among subjects in China, Singapore and Thailand reported a continuous abstinence rate of 38.2% at 24 weeks, where 4.8% of the subjects experienced severe adverse events, including insomnia and elevated liver enzymes [39]. Gastrointestinal disturbances were the most common adverse events in these studies [36,37,38,39]. In contrast, the continuous abstinence rate at 24 weeks in the current study was notably lower at 18.4%. Nevertheless, the adverse events reported were similar, dominated by gastrointestinal disturbances, with few neurological symptoms and rare cardiovascular adverse events. 

In the present study, incidences of suicide-related behaviour, such as ideation or self-harm behaviour, were not found. Nevertheless, altered behaviour was detected in one smoker who described starting to “isolate himself and becoming very quiet” after taking varenicline, which could be a presentation of depression. Meanwhile, the onset of psychosis symptoms in another smoker who experienced auditory hallucinations within a week of treatment is consistent with previously published cases [40]. Previous observational cohort studies and meta-analyses do not indicate a heightened risk of depression, suicidal ideation and self-harm among varenicline users [6,41,42]. Nevertheless, clinically important side effects, such as anxiety, irrational behaviour, aggression and restlessness [31], have been commonly reported, suggesting the need for structured pharmacovigilance regarding uncommon neuropsychiatric-related adverse events.

Although major adverse cardiovascular events are not reported in this study, population data consistently show the low occurrence of other cardiovascular diseases in smokers using varenicline [41,43]. Kortz et al. reported the incidence of events for ischaemic heart disease, cerebral infarct, heart failure and peripheral vascular disease as 23.4, 6.4, 2 and 4.8 incidences per 1000 patient years, respectively [41]. Three chest pain episodes requiring hospitalization were demonstrated in the current study. However, whether the smoker had progressed to myocardial infarction or heart failure was not investigated. The incidence of cardiovascular adverse events was relatively low among varenicline users, consistent with previous reports. Nevertheless, active surveillance of cardiovascular-related events is warranted, especially among smokers with such comorbidities.

The profile of other adverse events exhibited in this study is consistent with a previous Cochrane review [36]. The most frequently recorded adverse effect was nausea, which was mostly at mild-to-moderate levels and tended to subside over time [32]. The incidence of nausea in the varenicline groups was found to be dose-dependent, with higher prevalence in smokers taking a higher dose of 1 mg BD [44]. A case report demonstrated cessation of varenicline treatment due to nausea and insomnia intolerance [45]. In our study, as mentioned earlier, two smokers discontinued treatment due to intolerance to nausea and insomnia. Other common symptoms, such as dry mouth, constipation, stomach discomfort and flatulence, were not frequently observed. This could be because these symptoms were mild and did not warrant the attention of the smokers themselves, and the clinicians might not have proactively asked the patients regarding these symptoms.

This study had both strengths and limitations. To our knowledge, this is the first study to explore the association between varenicline adverse effects and withdrawal symptoms and smoking abstinence in Malaysia. There are several limitations worth mentioning. First, misclassification bias may be present since data collection was retrospective, based on previous records. Furthermore, the sample size was based on prevalence and not adequately powered to account for factors associated with smoking abstinence and adverse events. Hence, a future study with a larger sample size is needed to verify the findings of the current study. The types of concurrent medications was not captured, hence we could not rule out its confounding effects with the reported adverse events. As we excluded patients with missing data, the abstinence and adverse events rate might have been different if they were included. The absence of nicotine replacement therapy in several local health clinics limited our effort to make a useful comparison. While we followed up the adverse effects up to 24 weeks, future studies should examine the long-term side effects across the span of several years.

## 5. Conclusions

In conclusion, most of the subjects achieved smoking abstinence with varenicline. A longer duration of treatment, older age and the absence of comorbidities are significant predictors of successful abstinence. Despite the rare cases of cardiovascular- and neuropsychiatric-related adverse events, continuous surveillance and adverse drug reaction reporting is warranted.

## Figures and Tables

**Table 1:** Patient demographic characteristics (N = 114).

Demographic Characteristics	N (%)
**Age** (years), in mean ± SD	46 ± 14.4
**Weight** (kg), in mean ± SD	69.8 ± 14.99
**Gender**	
Male	104 (91.2)
Female	10 (8.8)
**Race**	
Malay	70 (61.4)
Chinese	17 (14.9)
Indian	27 (23.7)
**Comorbidities**	
Diabetes mellitus	30 (26.3)
Hypertension	26 (22.8)
Dyslipidaemia	19 (16.7)
Asthma	13 (11.4)
Coronary artery disease (CAD) (Stable CAD, acute coronary syndrome)	11 (9.6)
COPD	8 (7)
Cerebrovascular disease (stroke, transient ischemic attack, cerebrovascular accident)	3 (2.6)
Others	5 (4.4)

**Table 2:** Smokers’ characteristics, smoking history and behavior before varenicline treatment (N = 114).

	N (%)
**Age started smoking (years)**, mean ± SD	19.0 ± 5.9
**Number of cigarettes smoked per day (sticks)**, in mean ± SD before varenicline treatment	17.0 ± 11.3
**Duration of smoking (years)**, mean ± SD before varenicline treatment	26.6 ± 14.9
**Fagerstrom score**	
Score 0 to 3: low nicotine dependence	42 (36.8)
Score 4 to 5: moderate nicotine dependence	28 (24.6)
Score 6 to 10: high nicotine dependence	44 (38.6)
**Readiness to quit**	
Precontemplation	5 (4.4)
Contemplation	27 (23.7)
Preparation	38 (33.3)
Action	43 (37.7)
**Attempts to quit before varenicline treatment**	
Yes	79 (69.3)
No	35 (30.7)
**Triggering factors**	
After a meal	89 (78.1)
During social event/festive season	57 (50.0)
When feeling boring/drowsy	84 (73.7)
When feeling anxious/stressful	63 (55.3)
When colleagues/friends are smoking	82 (71.9)
When watching TV/videos/movies	31 (27.2)
In the bathroom	50 (43.9)
Staying with smokers	15 (13.2)

**Table 3:** Smoking abstinence and continuous abstinence among varenicline users. (N = 114).

Variables	N (%)
**Treatment duration** (week), in mean ± SD	8.8 ± 3.3
**Successful smoking abstinence (for at least 2 weeks)**	
Yes	68 (59.6)
No	46 (40.4)
**Continuous abstinence**	
≤12 weeks	22 (19.3)
13 to 16 weeks	12 (10.5)
17 to 20 weeks	13 (11.4)
24 weeks	21 (18.4)

**Table 4:** Adverse events and withdrawal symptoms after varenicline use (N = 114).

Adverse Events	N (%)
**Cardiovascular-related adverse events**	
Hospitalisation due to cardiovascular adverse events	2 (1.8)
Angina pectoris	1 (0.9)
**Neuropsychiatric-related adverse events**	
Altered behaviour	2 (1.8)
Auditory hallucination	1 (0.9)
**Other adverse events**	
Increased appetite	19 (16.7)
Dizziness	18 (15.8)
Dry mouth	12 (10.5)
Nausea	10 (8.8)
Abnormal dreams	9 (7.9)
Insomnia	9 (7.9)
Stomach discomfort	6 (5.3)
Vomiting	5 (4.4)
Flatulence	5 (4.4)
Diarrhea	4 (3.5)
Constipation	3 (2.6)
Abdominal distension	2 (1.8)
Sore throat	2 (1.8)
**Withdrawal symptoms**	**N (%)**
Fatigue	21 (18.4)
Headache	18 (15.8)
Irritability/restlessness	15 (13.2)
Anger/frustration	14 (12.3)
Change in sleep pattern	12 (10.5)
Impatience	9 (7.9)
Poor concentration	9 (7.9)
Palpitation	7 (6.1)
Change in bowel movement	5 (4.4)
Tremors	1 (0.9)

**Table 5:** Univariate and multivariate logistic regression analysis on predicting factors of smoking abstinence.

	Univariate Logistic Regression			Multivariate Logistic Regression		
Variables	Odds Ratio	Confidence Interval 95%	*p*-Value	Odds Ratio	Confidence Interval 95%	*p*-Value
**Age**	1.009	0.983–1.036	0.511			
**Gender**						
Female	Reference	Reference	Reference			
Male	1.537	0.419–5.641	0.517			
**Race**						
Malay	Reference					
Chinese	1.083	0.358–3.276	0.887			
Indian	0.549	0.224–1.346	0.190			
Comorbidities						
Without	Reference					
With	2.091	0.969–4.514	0.060			
**Age started smoking**	1.028	0.960–1.102	0.423			
**Number of cigarettes smoked per day**	1.010	0.977–1.045	0.554			
**Duration of smoking**	1.005	0.980–1.031	0.700			
**Fagerstrom score**						
High	Reference					
Low-to-moderate	0.890	0.412–1.924	0.767			
**Without quit attempt**						
With attempt	Reference					
Without attempt	0.727	0.325–1.627	0.438			
**Readiness to quit**						
Precontemplation and contemplation	Reference					
Preparation and action	1.500	0.655–3.433	0.337			
**Triggering factors**						
Situational factor	0.692	0.256–1.875	0.469			
Mood effect	0.838	0.303–2.318	0.733			
Peer influence	0.772	0.319–1.867	0.565			
**Treatment duration**	1.828	1.501–2.226	<0.001 *	1.828	1.501–2.226	<0.001 *
**Adverse events**						
Yes	Reference					
No	0.566	0.262–1.223	0.147			
**Withdrawal symptoms**						
Yes	Reference					
No	1.123	0.520–2.429	0.767			

* significant values (*p* < 0.05).

## Data Availability

The dataset used in this study can be obtained from the authors upon reasonable requests.

## References

[1] World Health Organization (2017). WHO Report on the Global Tobacco Epidemic, 2017: Monitoring Tobacco Use and Prevention Policies.

[2] World Health Organization (2019). WHO Global Report on Trends in Prevalence of Tobacco Use 2000–2025.

[3] Institute for Public Health (2015). National Health and Morbidity Survey 2015 (NHMS 2015): Vol. II: Non-Communicable Diseases, Risk Factors & Other Health Problems.

[4] Institute for Public Health (2012). Report of Global Adult Tobacco Survey (GATS) Malaysia 2011.

[5] Singh D., Saadabadi A. (2022). Varenicline. StatPearls.

[6] Cahill K., Stevens S., Perera R., Lancaster T. (2013). Pharmacological interventions for smoking cessation: An overview and network meta-analysis. Cochrane Database Syst. Rev..

[7] Wu P., Wilson K., Dimoulas P., Mills E.J. (2006). Effectiveness of smoking cessation therapies: A systematic review and meta-analysis. BMC Public Health.

[8] Kotz D., Brown J., West R. (2014). Prospective cohort study of the effectiveness of varenicline versus nicotine replacement therapy for smoking cessation in the “real world”. BMC Public Health.

[9] U. S. Food and Drug Administration (2009). FDA Adds Boxed Warnings to Varenicline and Bupropion. http://www.cardiobrief.org/2009/07/01/fda-adds-boxed-warnings-to-varenicline-and-buproprion/.

[10] U. S. Food and Drug Administration (2011). FDA Drug Safety Communication: Chantix® (Varenicline) May Increase the Risk of Certain Cardiovascular Adverse Events in Patients with Cardiovascular Disease. https://www.fda.gov/drugs/drug-safety-and-availability/fda-drug-safety-communication-chantix-varenicline-may-increase-risk-certain-cardiovascular-adverse.

[11] Anthenelli R.M., Benowitz N.L., West R., St Aubin L., McRae T., Lawrence D., Ascher J., Russ C., Krishen A., Evins A.E. (2016). Neuropsychiatric safety and efficacy of varenicline, bupropion, and nicotine patch in smokers with and without psychiatric disorders (EAGLES): A double-blind, randomised, placebo-controlled clinical trial. Lancet.

[12] Ware J.H., Vetrovec G.W., Miller A.B., Van Tosh A., Gaffney M., Yunis C., Arteaga C., Borer J.S. (2013). Cardiovascular safety of varenicline: Patient-level meta-analysis of randomized, blinded, placebo-controlled trials. Am. J. Ther..

[13] Tobacco Control Unit & FCTC Secretariat, Ministry of Health Malaysia (2016). Clinical Practice Guidelines on Treatment of Tobacco Use Disorder. https://www.moh.gov.my/moh/resources/Penerbitan/CPG/Respiratory/CPG_TobacoDisorder.pdf.

[14] Rigotti N.A., Pipe A., Garza D., Arteaga C., Benowitz N.L., Tonstad S. (2009). Efficacy and safety of varenicline for smoking cessation in patients with 52 cardiovascular disease: A randomized trial. J. Am. Coll. Cardiol..

[15] Ministry of Health Malaysia (2019). Quit Smoking Pharmacotherapy Guidelines.

[16] Hughes J.R., Keely J.P., Niaura R.S., Ossip-Klein D.J., Richmond R.L., Swan G.E. (2003). Measures of abstinence in clinical trials: Issues and recommendations. Nicotine Tob. Res..

[17] Heatherton T.F., Kozlowski L.T., Frecker R.C., Fagerström K.O. (1991). The Fagerström Test for Nicotine Dependence: A revision of the Fagerström Tolerance Questionnaire. Br. J. Addict..

[18] Watson D., Clark L.A., Chmielewski M., Kotov R. (2013). The value of suppressor effects in explicating the construct validity of symptom measures. Psychol. Assess..

[19] Lee Y.Y., Khoo S., Morris T., Hanlon C., Wee L.H., Teo E.W., Adnan Y. (2016). A mixed-method study of the efficacy of physical activity consultation as an adjunct to standard smoking cessation treatment among male smokers in Malaysia. SpringerPlus.

[20] Shafie A.A., Hassali M.A., Rabi R., Lee M.L. (2016). Treatment outcome assessment of the pharmacist-managed quit smoking clinic in Malaysia. J. Smok. Cessat..

[21] Wee L.H., West R., Bulgiba A., Shahab L. (2010). Predictors of 3-month abstinence in smokers attending stop-smoking clinics in Malaysia. Nicotine Tob. Res..

[22] Zainal M.A., Kadir H., Manaf R.A. (2017). Outcome and Predictors for Smoking Cessation in a Quit Smoking Clinic. Int. J. Public Health Res..

[23] Reid J.L., Hammond D., Boudreau C., Fong G.T., Siahpush M., ITC Collaboration (2010). Socioeconomic disparities in quit intentions, quit attempts, and smoking abstinence among smokers in four western countries: Findings from the International Tobacco Control Four Country Survey. Nicotine Tob. Res..

[24] Qiu D., Chen T., Liu T., Song F. (2020). Smoking cessation and related factors in middle-aged and older Chinese adults: Evidence from a longitudinal study. PLoS ONE.

[25] Joly B., Perriot J., d’Athis P., Chazard E., Brousse G., Quantin C. (2017). Success rates in smoking cessation: Psychological preparation plays a critical role and interacts with other factors such as psychoactive substances. PLoS ONE.

[26] Drovandi A.D., Chen C.C., Glass B.D. (2016). Adverse Effects Cause Varenicline Discontinuation: A Meta-Analysis. Curr. Drug Saf..

[27] Hajek P., McRobbie H., Myers Smith K., Phillips A., Cornwall D., Dhanji A. (2015). Increasing Varenicline Dose in Smokers Who Do Not Respond to the Standard Dosage: A Randomized Clinical Trial. JAMA Intern. Med..

[28] Chean K.Y., Goh L.G., Liew K.W., Tan C.C., Choi X.L., Tan K.C., Ooi S.T. (2019). Barriers to smoking cessation: A qualitative study from the perspective of primary care in Malaysia. BMJ Open.

[29] Kristina S.A., Vo Q.T., Dwinta E., Rahman F. (2018). Individual, social and psychological characteristics of smoking cessation behaviors: A systematic review. Glob. J. Health Sci..

[30] Levshin V., Slepchenko N. (2017). Determinants of smoking cessation and abstinence in a Russian smoking cessation center. Tob. Prev. Cessat..

[31] Ezat W.S., Selahuddeen A.A., Aljunid S.M., Zarihah Z. (2008). Pattern and predictors of smoking cessation among smokers attending smoking cessation clinics in Peninsular Malaysia. J. Community Health.

[32] Ho K.S., Choi B.W., Chan H.C., Ching K.W. (2016). Evaluation of biological, psychosocial, and interventional predictors for success of a smoking cessation programme in Hong Kong. Hong Kong Med. J..

[33] Ussher M., Kakar G., Hajek P., West R. (2016). Dependence and motivation to stop smoking as predictors of success of a quit attempt among smokers seeking help to quit. Addict. Behav..

[34] Saba M., Diep J., Bittoun R., Saini B. (2014). Provision of smoking cessation services in Australian community pharmacies: A simulated patient study. Int. J. Clin. Pharm..

[35] Dobrinas M., Blanc A.L., Rouiller F., Christen G., Coronado M., Tagan D., Schäli C. (2014). Clinical pharmacist’s role in implementing a smoking cessation intervention in a Swiss regional hospital: An exploratory study. Int. J. Clin. Pharm..

[36] Ebbert J.O., Hughes J.R., West R.J., Rennard S.I., Russ C., McRae T.D., Treadow J., Yu C.R., Dutro M.P., Park P.W. (2015). Effect of varenicline on smoking cessation through smoking reduction: A randomized clinical trial. JAMA.

[37] Bolliger C.T., Issa J.S., Posadas-Valay R., Safwat T., Abreu P., Correia E.A., Park P.W., Chopra P. (2011). Effects of varenicline in adult smokers: A multinational, 24-week, randomized, double-blind, placebo-controlled study. Clin Ther..

[38] Rennard S., Hughes J., Cinciripini P.M., Kralikova E., Raupach T., Arteaga C., St Aubin L.B., Russ C. (2012). Flexible Quit Date Study Group. A randomized placebo-controlled trial of varenicline for smoking cessation allowing flexible quit dates. Nicotine Tob. Res..

[39] Wang C., Xiao D., Chan K.P., Pothirat C., Garza D., Davies S. (2009). Varenicline for smoking cessation: A placebo-controlled, randomized study. Respirology.

[40] Ahmed A.I., Ali A.N., Kramers C., Härmark L.V., Burger D.M., Verhoeven W.M. (2013). Neuropsychiatric adverse events of varenicline: A systematic review of published reports. J. Clin. Psychopharmacol..

[41] Kotz D., Viechtbauer W., Simpson C., van Schayck O.C., West R., Sheikh A. (2015). Cardiovascular and neuropsychiatric risks of varenicline: A retrospective cohort study. Lancet Respir. Med..

[42] Cahill K., Lindson-Hawley N., Thomas K.H., Fanshawe T.R., Lancaster T. (2016). Nicotine receptor partial agonists for smoking cessation. Cochrane Database Syst. Rev..

[43] Gershon A.S., Campitelli M.A., Hawken S., Victor C., Sproule B.A., Kurdyak P., Selby P. (2018). Cardiovascular and neuropsychiatric events after varenicline use for smoking cessation. Am. J. Respir. Crit. Care Med..

[44] Nakamura M., Oshima A., Fujimoto Y., Maruyama N., Ishibashi T., Reeves K.R. (2007). Efficacy and tolerability of varenicline, an α4β2 nicotinic acetylcholine receptor partial agonist, in a 12-week, randomized, placebo-controlled, dose-response study with 40-week follow-up for smoking cessation in Japanese smokers. Clin. Ther..

[45] Arteaga M.T.L., Amo C., Morla E.M.S., Román M.S. (2011). Induced psychosis after withdrawal of varenicline: A case report. Acta Neuropsychiatr..

